# Evaluation of a systematic digitized training program on the effectivity of subgingival instrumentation with curettes and sonic scalers in vitro

**DOI:** 10.1007/s00784-020-03356-8

**Published:** 2020-05-30

**Authors:** Christian Graetz, Paula Fecke, Miriam Seidel, Anne Sophie Engel, Susanne Schorr, Johanna Sentker, Christof E. Dörfer, Sonja Sälzer

**Affiliations:** Clinic for Conservative Dentistry and Periodontology, School of Dental Medicine, Christian-Albrechts-University of Kiel, Arnold-Heller-Str. 3, Haus B, 24105 Kiel, Germany

**Keywords:** Scaling and root planing, Non-surgical periodontal therapy, Subgingival hard deposits, Training evaluation, Biofilm removal

## Abstract

**Objectives:**

Whereas the key role of subgingival instrumentation in periodontal therapy is well known, the influence of operators’ experience/training with different devices on treatment results is yet uncertain. Therefore, we assessed untrained undergraduate students, working on manikins, as to how effectively they learn to use curettes (GRA) and sonic scalers (AIR); hypothesizing that AIR will result in higher relative cleaning efficacy (RCE) than GRA.

**Material and methods:**

Before baseline evaluation (T0), 30 operators (9 males, 21 females) received a 2-h theoretical lesson for both instruments, followed by a 12-week period with a weekly digitized training program for 45 min. During three sessions (T1–T3), the operators had to instrument six equivalent test teeth with GRA and AIR. At T0–T3, treatment time, proportion of removed simulated biofilm (RCE-b), and hard deposits (RCE-d) were measured.

**Results:**

At T0, RCE-b was in mean(SD) 64.18(25.74) % for GRA, 62.25(26.69) % for AIR; (*p* = 0.172) and RCE-d 85.48(12.32) %/ 65.71(15.27) % (*p* < 0.001). At T3, operators reached highest RCE-b in both groups (GRA/AIR 71.54(23.90) %/71.75(23.05)%; *p* = 0.864); RCE-d GRA/AIR: 84.68(16.84) %/77.85(13.98) %; *p* < 0.001). Both groups achieved shorter treatment times after training. At T3, using curettes was faster (GRA/AIR 16.67(3.31) min/19.80(4.52) min; *p* < 0.001).

**Conclusions:**

After systematic digitized training, untrained operators were able to clean 70% of the root surfaces with curettes and sonic scalers.

**Clinical relevance:**

It can be concluded that a systematic digitized and interactive training program in manikin heads is helpful in the training of root surface debridement.

## Introduction

Periodontitis is described as a multifactorial inflammatory disease associated with dysbiotic plaque biofilms and characterized by progressive destruction of the tooth-supporting apparatus [1]. Whereas untreated periodontitis eventually leads to tooth loss, an adequate periodontal therapy achieves biological compatibility of the previously diseased root surfaces [2, 3], thereby, allowing reattachment of adjacent tissues [4–7]. To do so, nonsurgical mechanical elimination of biofilm and/or hard deposits (mineralized biofilm) with, e.g., hand instruments or powered scaler [8, 9] is the cornerstone of each periodontal therapy and until now without any equivalent alternative [10]. Hence, it is the first step in a comprehensive periodontal disease treatment plan and further surgical interventions are not needed in most cases [11]. However, an effective performance of mechanical plaque removal is a prerequisite and needs to be trained intensely. Usually, before treating in clinical conditions, manual skills of dental students are trained in manikin heads. The challenge is to correctly and effectively apply appropriate instruments on a dentition. Hence, such a systematic training should include repetitive practical procedures [12]. Hereby, the aim is to increase the students’ experience and effectiveness in removal of simulated biofilm/hard deposits as well as to minimize adverse impacts [13]. Although the artificial models do not simulate a perfect realistic situation, the training is an important step to shorten treatment time and improve results [14]. Furthermore, a direct visualization of the percentage of biofilm and hard deposit removal is only possible in an in vitro situation.

Therefore, the aim of the study was to assess the training effect of a new digitally supported education program with regard to efficacy as well as time for removal of simulated biofilm and of hard deposits, with different instruments on untrained undergraduate students. Our main hypothesis was that the training effect for sonic scalers (AIR) compared with curettes (GRA) is greater at the end of the observation time, and the effect will be determined by specific factors, e.g., tooth type or jaw.

## Material and methods

### Experimental setup

All operators had the same setup and options to choose their instruments for root surface instrumentation; (1) Gracey normal-shape curettes nos. 5/6, 7/8, 11/12, and 13/14 (American Eagle Instruments, Missoula, MT, USA) and (2) sonic scaler (Synea, W&H, Bürmoos, Austria) with air pressure and water-cooling (30 ml/min) as recommended by the manufacturer on level two, which means “medium” amplitude, combined with a straight, right and left curved slimline tip (1AP, 2APr, 2APl, W&H, Bürmoos, Austria) with round cross-section.

New instruments were used only at baseline (T0), for following visits the curettes were randomized to each operator, whereby prior to each test day (T1–T3), the curettes were controlled by a trained dental hygienist regarding sharpness and if necessary sharpened with an Arkansas stone and grinding oil. Similarly, each tip for the sonic device was controlled for their length as advised by the manufacturer or any possible destruction.

### Operators training program and instrumentation procedure

**Fig. 1:**
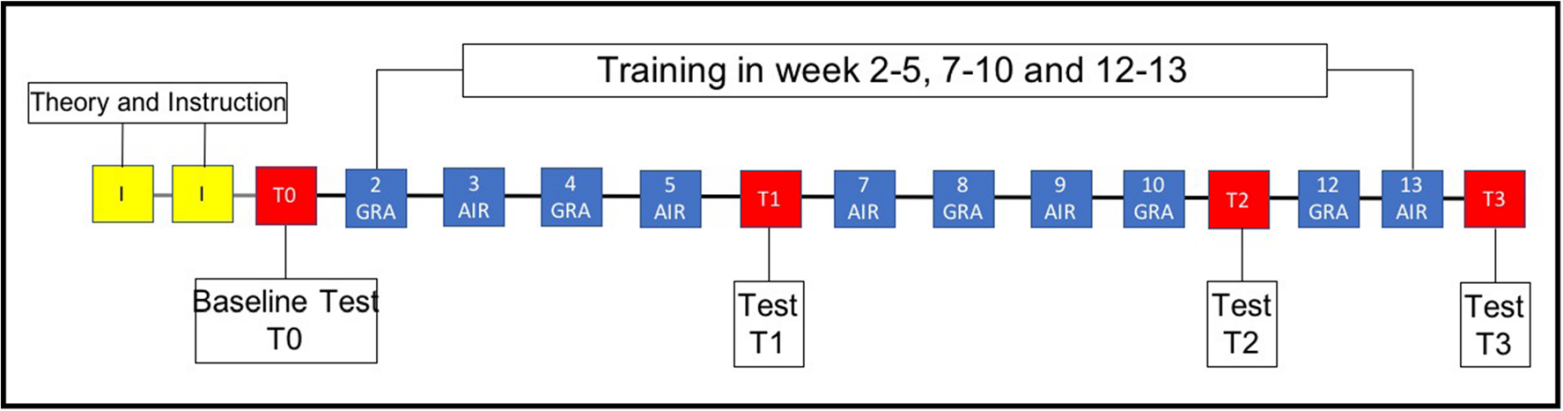
Training and test protocol: All operators trained curettes (GRA) and sonic scaler (AIR) 10 times in 14 weeks in a digitized learning program. At the outset of the study (before T0), operators received instructions and handling information (I) of the instruments in two lectures. Test teeth were evaluated at baseline (T0) and between the training weeks (T1, T2, and T3)

All participants received the same training program including two lectures of theoretical application information according to our clinical and the manufacture’s guideline as well as practical sessions in vitro before baseline test (Fig. 1). Afterwards, both groups of instruments were trained equivalently 10 times for 45 min over a 14-week period by using a digitally modified learning program (for AIR/GRA 24/32 working steps) based on a program introduced in 1994 [12]. Concomitant to the digitized training program the group was supervised and monitored through the education staff (3 board certified specialist of periodontology). In a previously published study [15], we could demonstrate that training of root surface instrumentation should not only focus on efficacy, but also on ergonomics. The digitized visualization program is supposed to support the teaching of the necessary working steps and ergonomic aspects separately for each instrument, and concomitantly reduces time effort for supervision by the staff. Partly by animated GIF or short video sequences the whole set up, technique and sitting position of the operator and patient are explained. Additionally, all participants were during training sessions clinically calibrated regarding the application pressure (3–5 N for GRA and < 1 N for AIR), but no measuring of the root surface destruction or roughness were done at the test days.

Before the study, at the beginning of the workshop all students of the 7th semester (*n* = 31) were asked to participate in the study, and after their written consent (*n* = 30), they received a 2-h theoretical introduction for both instruments and to the training program, but without practical exercises. One student, refused to take part in the study.

Participants were evaluated at baseline (T0) and between the training weeks at week 6 (T1), week 11 (T2) and week 14 (T3) while instrumenting 12 comparable test teeth with GRA (tooth 11, 14, 16, 31, 37, 45) and with AIR (tooth 21, 25, 17, 34, 36, 43). The instrument to begin with was randomized (Microsoft Excel 16, Microsoft Corporation, One Microsoft Way Redmond, WA, USA) for each test day and participant. Participants were asked to instrument the test teeth until they achieved subjective maximal elimination of simulated hard and soft deposits. Time for treating the predetermined six teeth per instrument was recorded for each participant.

### Manikin heads and test teeth

Both groups of instruments were performed in similar manikin heads, equipped with modified periodontitis models (Frasaco, Tettnang, Germany), exhibiting pronounced periodontitis with moderate to advanced horizontal bone loss, isolated and deep vertical pockets, resulting in teeth being differently difficult to instrument regarding both anatomy and accessibility. Mean (SD) pocket probing depth (PPD) was 5.8 (2.1) mm (range, 3–11 mm), separated for test teeth in GRA group (5.69 (2.01) mm (range, 3–9 mm)) versus AIR-group (5.67 (2.22) mm (range, 3–9 mm)). Gingival masks were fixed that no operator was able to lift the mask during the test trials.

Twelve teeth and seven single- and five multi-rooted teeth, were coated with a thin layer of transparent fluorescent varnish (Shiny White–Rival de Loop Young, Berlin, Germany) between the artificial cemento-enamel junction and the alveolar bone in order to simulate adhering plaque. Coating was performed by a reproducible and standardized procedure of dipping to provide similar thickness of the applied varnish [16]. The used varnish fluoresced with bright blue color when exposed to ultraviolet light. Gingival masks of persistently pliable silicon (Frasaco, Tettnang, Germany) were used to cover the models to ascertain instrumentation without visual control. Beside this simulation of biofilm, test-teeth were also randomly coated with commercial varnish (A-CK, Frasaco, Tettnang, Germany), modified for the ratio of varnish/thickener, to simulate adhering subgingival hard deposits. The hard deposits were located in spot size (diameter around 1.5 mm) at each tooth type at the same area by one investigator (P.F.) according to pictures taken during the first test. The varnish on the root surfaces was placed at a distance of at least 2 mm from the bottom of the pocket and 1 mm subgingival from the marginal gingiva, to simulate the pathologic circumstances as realistic as possible.

Artificial biofilm and hard deposits as well as teeth and gingival masks were renewed after each test day (T0–T3).

### Planimetric evaluation

**Fig. 2:**
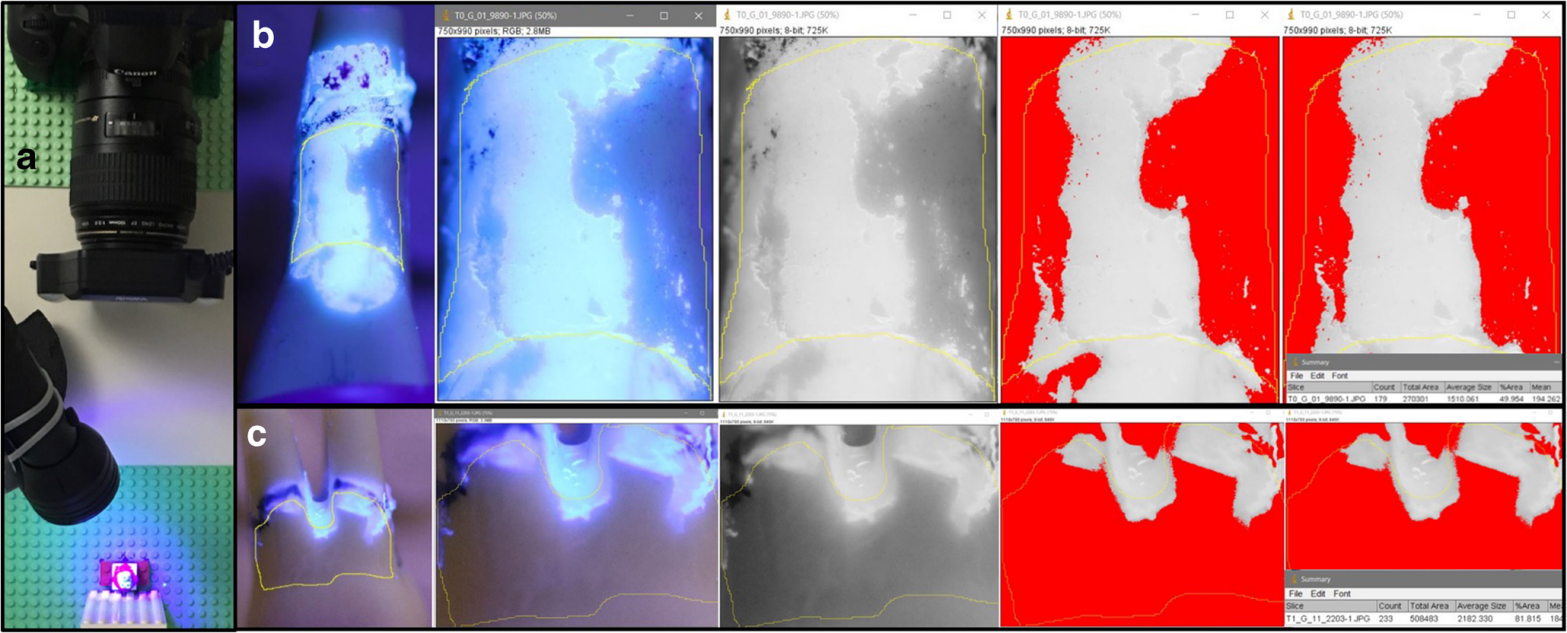
a Setup for photographic documentation. The test teeth were reproducibly fixed in the test blocks in order to photograph the cleaned root surface. **b** Standardized photographic documentation exemplified for tooth 11 of the buccal root surface and **c** the oral root surface of tooth 37. Subsequently the cleaned surfaces were evaluated in percent by a digital image subtraction (Image J, NIH, Bethesda, USA) in order to calculate the relative cleaning efficacy of simulated biofilm (RCE-b in %)

After each test day T0–T3, the effectivity of instrumentation was planimetrically assessed. Instrumented teeth were mounted on standardized prepared plastic units (Lego GmbH, Grasbrunn, Germany), individually fitting to each of the 12 teeth. Plastic units with mounted teeth were then fixed on the camera table in a reproducible position. Following, teeth were submitted to ultraviolet light (UV-A, 350–370 nm), and one image of each of the four tooth surfaces (mesial, distal, vestibular, oral) (in total 1728) was taken using a camera with a 100-mm macro-zoom (Canon EOS D30, Tokyo, Japan). The images were used to subsequently perform an evaluation of the cleaned surface area by digital image subtraction (Image J, NIH, Bethesda, USA) to calculate the relative cleaning efficacy of simulated biofilm removal subgingival (RCE-b in %) (Fig. 2). For multi-rooted teeth with furcation involvement (16, 37, 17, 36), no evaluation of the furcation area was done (please see Fig. 2c for details). The relative cleaning efficacy of hard deposits (RCE-d) was assessed through one investigator (P.F.) by counting the non-removed hard deposits on reproducible images of each tooth site.

### Outcomes

As the primary outcome, the percentage of removed artificial biofilm and hard deposits were determined. Secondary outcomes were treatment time, difference between test days and instruments according to the cleaning effectiveness as well as operators’ experience reports.

#### Relative cleaning efficacy of simulated biofilm removal—RCE-b in%

Measuring RCE as the difference of the area with simulated biofilm before and after cleaning the four different root surface areas of each test tooth (n = 48 of 12 test teeth) and each operator (n = 30), separately for the two types of instrumentation (GRA vs. AIR) and the four test days (T0-T3).

#### Relative cleaning efficacy of hard deposit removal—RCE-d in%

Measuring RCE-d as the difference of the number of simulated hard deposits before and after cleaning root surface areas of all test teeth (n = 12) and operators (n = 30), separately for the two types of instrumentation (GRA versus AIR) and the four test days (T0 T3). It should be noted in comparison to RCE-b, for RCE-d only the percentage of the number of removed hard deposits compared with the total number of deposits was evaluated. It was no evaluation undertaken to measure the size of the cleaned area as hard deposits were only simulated in a small spot.

#### Treatment time in min

Accordingly, time for treating test teeth (n = 6) per group of instruments (GRA versus AIR) was measured separately for each operator (n = 30) at the four test days (T0 -T3). Duration for changing instruments was taken into consideration.

The investigator (J.S.) was blinded to the used instruments and test days. For cleaning efficacy, two levels were defined. For level one, ≥70% of simulated biofilm or hard deposits had to be removed, for level two ≥80%. Not adequate was set as 0-69% of removed simulated biofilm or hard deposits.

### Questionnaire

At each test day (T0 -T3), operators answered a questionnaire to estimate their own effectiveness in removing simulated biofilm/hard deposits for each group of instruments as well as questions according gender, age and previous vocational training only at T0 (closed-ended question).

### Statistical analysis

According to the sample size calculation with data of a comparable investigation [12] we found n = 175 teeth per group at each test day as sufficient to detect under five percent difference for removed hard deposits between the groups of instruments (power of 80%).

Randomization, means, percentages and standard deviations were calculated using Microsoft Excel (Microsoft Excel 16, Microsoft Corporation, One Microsoft Way Redmond, WA, USA). Values were mostly ordinal (yes/no) and with the use of Microsoft Excel, crosstabs have been drawn up. Data were entered into SPSS Statistics (SPSS Statistics 24, IBM, Chicago, IL, USA). Normal distribution of artificial biofilm, residual hard deposits and treatment time were tested by Kolmogorov-Smirnov, Shapiro-Wilk as well as Chi Quadrat Test. The artificial biofilm at the two test days (T2 and T3) was not normally distributed (p > 0.0001). Artificial biofilm removal, therefore, were tested with non-parametric tests. All tests were two-sided; statistical significance was assumed p ≤ 0.05 and adjusted with the Bonferroni method for multiple comparison (p = 0.05 / 6 = 0.0083). Associations between variables were analyzed using Pearson correlation and Spearman's rank correlation coefficient analysis.

## Results

### Operators’ characteristics

All 30 operators were in the seventh semester at Kiel dental school (female/male 21/9) and in mean (SD) 25.50(4.11) years of age (range 21-42). Eleven had a previous education in dentistry e.g. dental assistants or technician (female/male 7/4).

### Removal of simulated biofilm and hard deposits—efficacy and learning curve

**Fig. 3:**
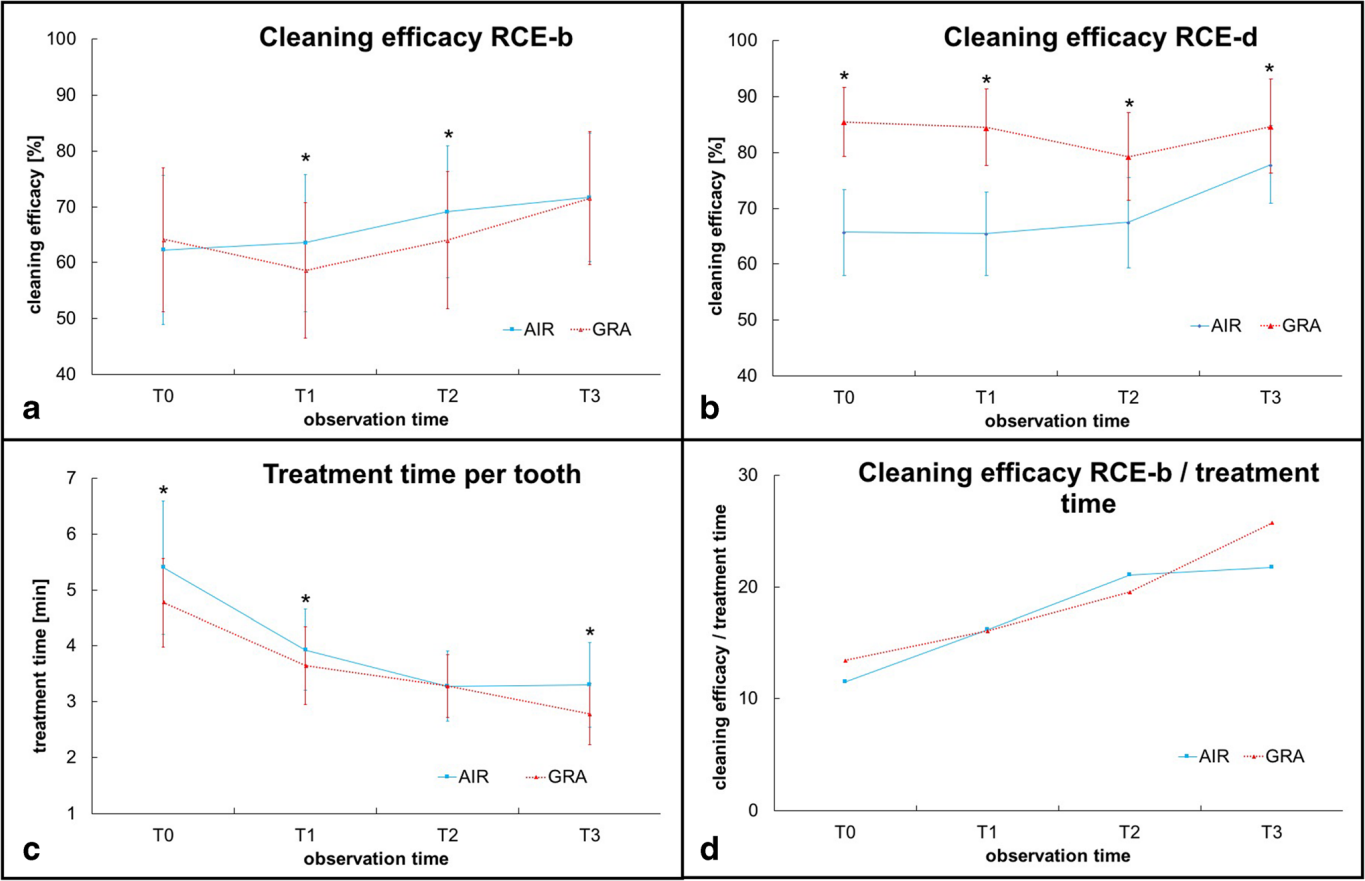
Cleaning efficacy **a** for removal of simulated biofilm (RCE-b in %), **b** removal of hard deposits (RCE-d in %), **c** the treatment time per tooth (in min), and **d** the calculated cleaning performance (RCE-b/treatment time per tooth) for sonic instruments (AIR) versus curettes (GRA) at T0–T3. (*p ≤ 0.05)

In general, the learning curve of the yet-untrained operators begins with a nearly equal efficacy level for GRA of 63.18(25.74) % and for AIR of 62.25(26.69) % RCE-b at T0 (*p* = 0.172). In the GRA-group RCE-b decreases until T1 to 58.62(24.18) % (*p* < 0.001) and subsequently improves significantly at T2 (RCE-b 64.05(24.51) %, *p* < 0.001) and reaches highest efficacy at T3 (RCE-b 71.54(23.90) %, *p* < 0.001) (Table 1, Fig. 3). For AIR-group, we found a different performance with continually improvement of the RCE-b values after T0 until T3. At T1, the operators showed a 63.56(24.55) % efficacy without significant changes between T0 and T1 (*p* = 0.203), whereas at T2 (69.17(23.55) % and T3 (71.75(23.05) % efficacy significantly improved (T1–T2: *p* < 0.001 and T2–T3: *p* = 0.001) (Table 1, Fig. 3). Despite the different progression during the training phase, all operators removed simulated biofilm without significant difference between both groups of instruments at the final evaluation (T3: *p* = 0.864).

**Table 1:** Efficacy of removed simulated biofilm (RCE-b in %) and hard deposits (RCE-d in %) with hand (GRA) or airscaler (AIR) and the treatment time (in min) for all 12 test teeth at four different test days (T0-T3). Average values (SD) and p-values (^a^Bonferroni adjustment (p ≤ 0.0083)). *(N of RCE-b in total 720 and N of RCE-d in total 420)

**Mean (SD)**															
**Test day**	**RCE-b in %**					**RCE-d in %**					**Treatment time in min per 6 test teeth in min**				
	N of GRA*	N of AIR*	**GRA**	**AIR**	**Differences between groups**	N of GRA*	N of AIR*	**GRA**	**AIR**	**Differences between groups**	N of GRA*	N of AIR*	**GRA**	**AIR**	**Differences between groups**
T0	672	712	64.18(25.74)	62.25(26.69)	p = 0.172	386	414	85.48(12.32)	65.71(15.27)	p < 0.001	30	27	28.63(4.77)	32.41(7.17)	p < 0.001
T1	700	712	58.62(24.18)	63.56(24.55)	p < 0.001	407	414	84.52(13.58)	65,48(14.90)	p < 0.001	30	30	21.87(4.18)	23.57(4.34)	p < 0.001
T2	640	680	64.05(24.51)	69.17(23.55)	p < 0.001	374	398	79,26(15.71)	67.49(16.11)	p < 0.001	30	30	19.67(3.40)	19.67(3,78)	p = 1.000
T3	660	704	71.54(23.90)	71.75(23.05)	p = 0.864	378	413	84.68(16.84)	77.85(13.98)	p < 0.001	30	30	16.67(3.31)	19.80(4.52)	p < 0.001
**Differences between test days T0–T3 for RCE-b, RCE-d and treatment time**															
			***p*** **values**^**a**^	***p*** **values**^**a**^				***p*** **values**^**a**^	***p*** **values**^**a**^				***p*** **values**^**a**^	***p*** **values**^**a**^	
T0 vs. T1			*p* < 0.001	*p* = 0.203				*p* = 0.257	*p* = 0.750				*p* < 0.001	*p* < 0.001	
T0 vs. T2			*p* = 0.627	*p* < 0.001				*p* < 0.001	*p* = 0.274				*p* < 0.001	*p* < 0.001	
T0 vs. T3			*p* < 0.001	*p* < 0.001				*p* = 0.417	*p* < 0.001				*p* < 0.001	*p* < 0.001	
T1 vs. T2			*p* < 0.001	*p* < 0.001				*p* < 0.001	*p* = 0.012				*p* < 0.001	*p* < 0.001	
T1 vs. T3			*p* < 0.001	*p* < 0.001				*p* = 0.885	*p* < 0.001				*p* < 0.001	*p* < 0.001	
T2 vs. T3			*p* < 0.001	*p* = 0.001				*p* < 0.001	*p* < 0.001				*p* < 0.001	*p* = 0.393

Considering hard deposits (RCE-d), the learning curves in the AIR-group are comparable with RCE-b. The comparison of RCE-d of both groups of instruments demonstrates significant superiority of the GRA-group at all time points (T0: GRA 85.48(12.32) % versus AIR 65.71(15.27) % efficacy; *p* < 0.001 and T3: GRA 84.68(16.84) % versus AIR 77.85(13.98) % efficacy; *p* < 0.001). RCE-d values for AIR at T0 and T1/T2 (65.48(14.90) %, *p* = 0.750/67.49 (16.11) %, *p* = 0.012 (adjusted to Bonferroni)) do not differ significantly. The improvement between T0 and T3 for RCE-d in the AIR-group is significant ((77.85(13.98) %, *p* < 0.001) (Table 1). The learning performance for GRA had a different pattern; after equal efficacy at T0 to T1 (84.52(13.58) %, *p* = 0.257) the lowest efficacy was found at T2 (79.26(15.71) %, *p* < 0.001) and increased again until T3 (84.68(16.84) %, *p* < 0.001). The difference between T0 and T3, however, was not significant (*p* = 0.417) (Table 1, Fig. 3).

Before the beginning of the training phase, for both groups of instrument no significant difference for removing simulated biofilm in the upper jaw versus the lower jaw were measured (GRA/AIR: *p* = 0.011 (adjusted to Bonferroni)/*p* = 0.367) (Table 2). At T3, both groups of instruments were able to reach a significant higher efficacy in the lower (GRA/AIR 68.88(23.36) %/70.05(23.18) %) versus upper jaw (GRA/AIR 74.23(24.18) %/AIR 73.39(22.38) %) (all groups T0–T3: *p* ≤ 0.001) without significant differences between the groups of instruments (upper jaw/lower jaw: *p* = 0.514/*p* = 0.638). For removal of hard deposits at T3 we found compared with RCE-b in the AIR-group, a higher efficacy in the upper jaw versus lower jaw (83.68(19.76) %/74.26(12.11) %, *p* < 0.001), but for GRA, results were nearly similar (95.95(11.52) %/(93.14(11.28) %, *p* = 0.311). Contrary to the training effect in simulated biofilm removal in both groups, an improvement in hard deposit removal was only observed for AIR.

**Table 2:** Efficacy of removed simulated biofilm (RCE-b in %) and hard deposits (RCE-d in %) with hand (GRA) or airscaler (AIR) at test days T0, T1, T2, and T3 separated for jaw (upper and lower) and tooth type (front teeth, premolar, and molar). Average values (SD) and *p* values (^a^Bonferroni adjustment (*p* ≤ 0.0083)). Data for RCE-d on tooth level were not available (n.a.)

**Mean (SD)**											
	**Test day**	**GRA**				**AIR**				**Differences between GRA vs. AIR**	
				**Differences between jaws**				**Differences between jaws**			
		**RCE-b**	**RCE-d**	**RCE-b**	**RCE-d**	**RCE-b**	**RCE-d**	**RCE-b**	**RCE-d**	**RCE-b**	**RCE-d**
Upper jaw	T0	66.63 (23.91)	92.95 (10.80)	*p* = 0.011	*p* = 0.822	63.16 (25.61)	71.66 (26.82)	*p* = 0.367	*p* = 0.099	*p* = 0.065	*p* < 0.001
	T1	56.49 (24.0)	84.01 (15.1)	*p* = 0.022	*p* = 0.140	66.49 (22.50)	71.90 (25.29)	*p* = 0.001	*p* = 0.049	*p* < 0.001	*p* = 0.028
	T2	61.67 (24.02)	87.68 (17.53)	*p* = 0.018	*p* = 0.586	68.52 (22.69)	74.87 (22.14)	*p* = 0.473	*p* = 0.055	*p* < 0.001	*p* = 0.018
	T3	68.88 (23.36)	95.95 (11.52)	*p* = 0.004	*p* = 0.311	70.05 (23.18)	83.68 (19.76	*p* = 0.055	*p* = 0.030	*p* = 0.514	*p* = 0.005
Lower jaw	T0	61.60 (27.33)	92.38 (16.66)			61.35 (27.70)	62.37 (14.25)			*p* = 0.907	*p* < 0.001
	T1	60.69 (24.21)	90.0 (13.62)			60.63 (26.15)	61.41 (13.19)			*p* = 0.979	*p* < 0.001
	T2	66.26 (24.80)	85.19 (16.26)			69.81 (24.39)	64.98 (15.20)			*p* = 0.061	*p* < 0.001
	T3	74.23 (24.18)	93.14 (11.28)			73.39 (22.83)	74.26 (12.11)			*p* = 0.638	*p* < 0.001
		**RCE-b**	**RCE-d**	**Differences between test day for RCE-b**^**a**^		**RCE-b**	**RCE-d**	**Differences between test day for RCE-b**^**a**^		**RCE-b**^**a**^	**RCE-d**
**Front teeth**	T0	58.60 (27.22)	n.a.	T0–T1: *p* = 0.157	T1–T3: *p* ≤ 0.001	65.08 (26.59)	n.a.	T0–T1: *p* = 0.298	T1–T3: *p* = 0.089	*p* = 0.010	n.a.
	T1	55.23 (25.10)		T0–T2: *p* = 0575	T2–T3: *p* ≤ 0.001	66.74 (23.14)		T0–T2: *p* = 0.321	T2–T3: *p* = 0.207	*p* < 0.001	
	T2	60.39 (27.28)		T0–T3: *p* ≤ 0.001		67.42 (23.13)		T0–T3: *p* = 0.013		*p* = 0.004	
	T3	71.14 (26.36)		T1–T2: *p* = 0.064		69.19 (22.59)		T1–T2: *p* = 0.958		*p* = 0.401	
**Pre-molar**	T0	67.36 (25.22)	n.a.	T0–T1: *p* = 0.235	T1–T3: *p* = 0.001	65.33 (25.42)	n.a.	T0–T1: *p* = 0.010	T1–T3: *p* ≤ 0.001	*p* = 0.388	n.a.
	T1	65.21 (24.55)		T0–T2: *p* = 0.490	T2–T3: *p* = 0.211	70.18 (20.05)		T0–T2: *p* ≤ 0.001	T2–T3: *p* = 0.017	*p* = 0.017	
	T2	68.05 (23.08)		T0–T3: *p* = 0.048		78.83 (19.56)		T0–T3: *p* ≤ 0.001		*p* < 0.001	
	T3	71.35 (22.09)		T1–T2: *p* = 0.264		81.28 (19.0)		T1–T2: *p* ≤ 0.001		*p* < 0.001	
**Molar**	T0	66.69 (23.72)	n.a.	T0–T1: *p* ≤ 0.001	T1–T3: *p* ≤ 0.001	56.42 (27.14)	k	T0–T1: *p* = 0.201	T1–T3: *p* ≤ 0.001	p < 0.001	n.a.
	T1	55.49 (21.56)		T0–T2: *p* = 0.017	T2–T3: *p* ≤ 0.001	53.99 (26.91)		T0–T2: *p* = 0.012	T2–T3: *p* = 0.028	*p* = 0.504	
	T2	64.01 (22.38)		T0–T3: *p* = 0.040		61.53 (24.32)		T0–T3: *p* ≤ 0.001		*p* = 0.258	
	T3	72.08 (23.35)		T1–T2: *p* ≤ 0.001		64.95 (24.10)		T1–T2: *p* ≤ 0.001		*p* = 0.001	

On tooth-level, at T3 in the AIR group, most simulated biofilm was removed in premolars compared to molars and front teeth (*p* < 0.001), but there are no such differences for the GRA group in RCE-b (Table 2). Using GRA in molars lead to significant better RCE-b results (*p* = 0.001) compared with AIR, whereas in premolars efficacy was higher in the AIR group (81.28(19.0) %; GRA 71.35(22.09); *p* < 0.001).

### Removal of simulated biofilm and hard deposits—treatment time

**Fig. 4:**
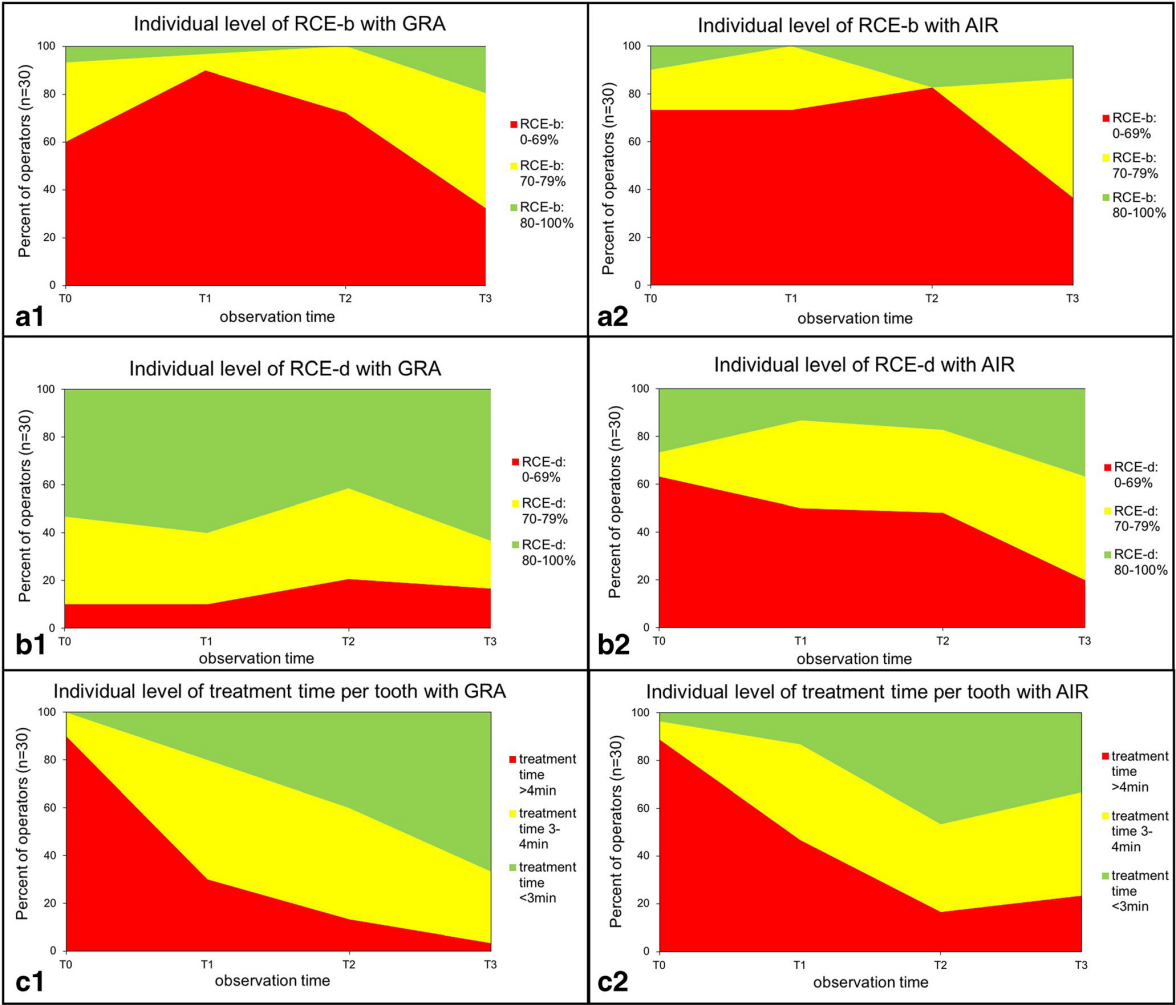
**a** Illustration of the number of operators (left side for gracey curettes (GRA) and right side for sonic scaler (AIR) per test day with cleaning efficacy of simulated biofilm removal (RCE-b) of 0–69%, 70–79%, and 80–100% and **b** for the efficacy of hard deposit removal (RCE-d), respectively. **c** Treatment time per tooth (categorized in green < 3 min, yellow 3–4 min, and red > 4 min) is illustrated both groups of instruments (GRA versus AIR).

With only one exception at T2 (*p* = 1.000), the treatment time for the six teeth per group was always shorter with GRA (Table 1, *p* < 0.001). At the final evaluation (T3), the calculated time per tooth was 2.78 (0.55) min per tooth for GRA (AIR 3.30(0.75) min). As illustrated in Fig. 3c, from baseline T0 until T3 the treatment time per tooth with curettes decreased around 2.0 min (AIR 2.1 min) (both: *p* < 0.001) and the majority of all thirty operators in both groups of instruments treated under 4 min (GRA: *n* = 29 and AIR: *n* = 23) (Fig. 4c). All operators reduced their own treatment time during the training phase with curettes, whereas with sonic scalers two operators failed to improve their own treatment time between T0 and T3. Regarding the performance as efficacy of RCE-b over treatment time, a continuous improvement from T0 to T3 can be observed for GRA and AIR (Fig. 3d).

### Removal of simulated biofilm and hard deposits—operators influence and self-assessment

At baseline T0, 60% of all operators in the GRA group were unable to reach an efficacy of RCE-b of 70% (73.33% of the AIR group). After the systematic training phase at T3 in both groups of instruments, this number of operators could be reduced to 33.33% (GRA) and 36.67% (AIR). For RCE-d, the learning curve was similar. Accordingly, at T0, no proband in the AIR group was able to remove all hard deposits (GRA 26.67%), but 10% at T3 (GRA 36.67%). Further changes in individual efficacy levels of RCE-b and RCE-d are shown in Fig. 4.

Furthermore, 18 operators showed higher efficacy to remove simulated biofilm with curettes at T3 versus T0 (AIR 24 operator) and 12 operators a lower efficacy (AIR 6 operators), respectively. For removal of hard deposits, 13 operators in the GRA group improved their own efficacy (AIR 22 operators), and 12 operators deteriorated (AIR 6 operators). Five operators showed similar results at T0 and T3 for RCE-d (AIR 2 operators).

At all test days, the operators self-estimated their efficacy of simulated biofilm removal (RCE-b) in both groups of instruments equally high with 60%-70% (Table 3).

**Table 3:** Average values (SD) of efficacy of removed simulated biofilm (RCE-b) and self-estimated (in %) by the operators for Gracey curettes (GRA) and for sonic scaler (AIR).

	GRA		AIR	
	RCE-b	Self-estimated RCE-b after	RCE-b	Self-estimated RCE-b
T0	64.18(25.74)	70.0(9.88)	62.25(26.69)	64.79(10.52)
T1	58.62(24.18)	65.3(10.20)	63.56(24.55)	65.21(8.94)
T2	64.05(24.51)	67.50(8.14)	69.17(23.55)	63.33(10.80)
T3	71.54(23.90)	72.08(11.04)	71.75(23.05)	69.17(11.06)

## Discussion

In a consensus report of the 1st European Workshop on Periodontal Education [13] for the undergraduate, dental curriculum and under supporting competences is recommended that dentists must be competent in “ … undertaking supra-gingival and sub-gingival scaling and root surface debridement, using both powered and manual instrumentation including stain removal and prophylactic procedures … ”. According to the common three levels of competence-based curricula, this corresponds to the highest level, as students should have a “ … theoretical knowledge and understanding of the subject together with an adequate clinical experience to be able to resolve clinical problems encountered, independently or without assistance … ” [13]. Although the necessity of theoretical and practical training within the framework of curricular education is indisputable, the key question remains on how to gain adequate clinical experience.

We could demonstrate in the current investigation that after a theoretical demonstration alone, untrained students were able to reach an efficacy to remove subgingival simulated biofilm (RCE-b) in mean of 64% (GRA) or 62% (AIR), and after 6 months of training once a week for 45 Minutes, they reached 71.5% (GRA) and 72% (AIR), corresponding to a significant improvement of 7.5%/10% (*p* ≤ 0.001). Interestingly, at T0, 40% of all 30 students already achieved an efficacy of over 70% for RCE-b with curette versus 27% only with AIR. During the training phase, more than half of all operators could improve their own efficacy to remove simulated biofilm. Though, more operators were able to reach this improvement by sonic scaler (GRA/AIR 18/24 operators).

For removal of hard deposits, we found only 13 operators, which improved their own efficacy using curettes between T0 and T3 versus 22 operators using sonic scalers. The observed superior efficacy at the beginning using GRA and the concomitant lack of improvement (T0 vs T3 for GRA 85.5% vs. 85%; *p* = 0.417) as well as the lower amount of operators demonstrating an improvement might be due to a structured training program on manikin heads teaching the application of a universal curette/scaler half a year before. This knowledge might now be transferred to the application of special curettes. On the other hand, they were totally inexperienced and not familiar with powered devices. Previous studies on undergraduate students, less-experienced or even experienced operators observed a similar phenomenon already [12, 16].

It does not seem to be surprising that the evaluated undergraduate students spend much more time for root surface debridement per tooth at the beginning of the training program (Table 2). Removal of invisible biofilm (subgingival) is difficult for less-experienced operator as they are yet not well trained in their tactility profound knowledge of the complex root morphology is missing. This leads to a significant increase of the results for RCE-b in the AIR-group already after a short time (T0–T1) and with a time delay also in the GRA-group (T0–T2). The time delay for GRA might be explicable by the more complex handling.

At T3, we found 85% of all operators achieved an efficacy of 70% for RCE-b (AIR 87%). For RCE-b, we failed to show significant differences between both groups of instruments before (T0) and after training (T3).

### Treatment time

Contrary to older position paper, in which powered devices were often described as more effective while, e.g., saving treatment time compared with hand instruments [17] which could not be supported by the results of our study. However, differences between a manikin model and a real clinical situation should be kept in mind. Untrained operators needed in the beginning of their systematic training phase independent of the instrument used nearly five Minutes per tooth improving to about 3 min at T3, favoring GRA at both time points (Table 1, *p* < 0.001). Therefore, we have to reject one part of our hypothesis that less-experienced operators after 6 months of training with sonic scaler will neither reach a higher efficacy nor save treatment time compared with curettes. We can only hypothesize on these differences. In the present study, the amplitude for the sonic scaler was fixed at medium level as advised by the manufacturer, but power adjustment of powered devices will influence the results significantly [18, 19]. However, these findings are in line with previous investigations [12, 16]. Our research underlines the recommendation for a comprehensively training of all powered devices, whose often times supposed perceived as simpler than that for hand instruments [20].

### Self-estimation

The operators of the current study could, however, estimate their own efficacy and treatment time more realistically compared with previous investigations for both instruments [21]. The training took the same amount of time, but our digitized training was supported by less supervisors (here with a ratio of 1:10). Nevertheless, the influence of motivation for the training seems to be enormous [21]. Motivated students (e.g., prizes for good performance, atmosphere of competition and enthusiasm during the whole training program) can achieve about 25% higher efficacy in removal of simulated biofilm compared with a group without motivational incentives [21]. Furthermore, motivation helped to estimate the efficacy more precisely and will greatly influence learning of effective root debridement [21]. Future studies could evaluate further developed digitized training programs with, e.g., personalized digital animation and immediate feedback function.

Furthermore, magnifying loupes might be of major importance already during training. They increase visual acuity and can therefore improve clinical outcomes in dentistry [22–24]. Despite the potential advantages in wearing loupes, in the current investigation their use was allowed, but not obligatory. However, the benefit of loupes during periodontal treatments is controversially discussed in literature [23–27], although a better view into the sulcus can be assumed.

### Limitations

For successful periodontal therapy, a major goal besides effective removal of biofilm and hard deposits is not to damage the root surface [28]. This outcome was however not assessed in the present study. According to our previous in vitro investigations focusing solely on simulated biofilm [15, 16] or on hard deposits [27], it might be helpful to consider the goal of the treatment for unexperienced operators. Thus, given the great variety of scope of interest and study designs, all comparisons between studies require caution [16, 19, 28–34]. Further on, due to the character of the in vitro simulation, e.g., using varnish to simulate the subgingival biofilm and hard deposits on the root surface of plastic teeth does not allow transferring our findings to clinical settings in general [29]. Using varnish to simulate subgingival biofilm and hard deposits allowed reproducible and standardized conditions in vitro, but the location of artificial biofilm under the simulated hard deposits as done in the current investigation is not comparably with a clinical situation. In vivo the development of hard deposits is a dynamic process that starts with a non-mineralized biofilm, which eventually calcifies. Nevertheless, it can also be seen that it does not matter whether there is a biofilm under or above a hard deposit as both have to be removed. Complete removal of hard deposits inevitably correlates with simultaneous removal of biofilm. Further, we failed to sub-analyze results of removed simulate hard deposits on jaw/tooth level, as we calculated only the percentage of the number of removed deposits compared with the total number of deposits due to technical reasons. Also, we cannot quantify possible effects of the used gingival masks, with a possible limited penetration of curettes into the sulcus and also possible damping of oscillating instruments [12, 16, 29, 35]. Furthermore, the morphology of the jaw and the tooth type are likely to have influenced our results as mentioned before. The furcation area of the involved molars was not assessed in this study, which might explain, why AIR failed to show better cleaning efficacy in molars as we assumed [17], whereas another in vitro investigation showed good accessibility when the horizontal pocket depth was less than 2 mm for both type of instruments [36]. Furthermore, as the tests were performed within one periodontitis model, the test teeth were not exactly corresponding but much with regard to type of tooth (front teeth, premolar, molar) in the upper and lower jaw and the profile of the used periodontitis models for both groups of instruments (AIR all quadrants vs. GRA: right upper and both lower quadrants). Therefore, we failed to control for possible confounder of handedness [37]. The analyzed teeth were selected to be comparable in terms of position (tooth type) and simulated probing depth/loss of attachment. Although, there is a difference in the location of the teeth between the two groups of instruments for premolars (teeth 14, 25 in the upper jaw and 34, 45 in the lower jaw), the morphology of the roots of all premolars are comparable (no furcation involvement).

Additionally, the haptic of the plaster material could as well have influenced our results due to the difficulties of simulating realistic bone morphology and haptic. However, the presented in vitro analyses allow investigating defined and clinically not measurable parameters under reproducible conditions, thereby increasing the sensitivity of the comparisons. Last but not least, the authors want to point out that the present study focused on the evaluation of the modified training program in routine teaching procedure and not to the best possible effectiveness of removal of simulated biofilm or hard deposits of undergraduate students.

Future studies should investigate during routine students’ workshops the combination of sonic scalers and curettes versus ultrasonic and subgingival air polishing with non-abrasive powder under clinical setting [10]. By teaching all these treatment methods for periodontal non-surgical therapy in dental school routine, it can be presumed that the next generation of dentists will work with the highest effectiveness concomitant with the lowest presumed number of side-effects. However, this was not evaluated in the current study. Therefore, all efforts should be done to improve the training programs and lectures in periodontology.

## Conclusions

Within the limitations of the present study, it can be concluded that a systematic digitized and interactive training program in manikin heads is helpful in the training of root surface debridement in less-experienced operators. After such training, the participants are able to remove simulated biofilm with an efficacy of more than 70% in mean and nearly 80% of the hard deposits. The performance as efficacy over treatment time improved continuously for both groups of instruments. Future studies should evaluate the interaction of direct digital feedback systems and user motivation during training.
